# Multiple mosquito species are potential competent vectors of avian malaria in the UK

**DOI:** 10.1017/S0031182026102303

**Published:** 2026-07

**Authors:** Jenny Claire Dunn

**Affiliations:** 1 School of Life and Environmental Sciences, Joseph Banks Laboratories, University of Lincoln, Lincoln, UK; 2 School of Life Sciences, Keele University, Newcastle, Staffordshire, UK

**Keywords:** Culicidae, haemosporidian, transmission, vector

## Abstract

Avian *Plasmodium, Haemoproteus* and *Leucocytozoon* (Apicomplexa) are common parasites of birds that are highly prevalent around the globe. Despite their broad impacts on avian hosts, remarkably little is known about their transmission ecology, including the identity of competent vector species. Both *Plasmodium* and *Haemoproteus* are known to detrimentally impact mosquito survival but have generally been neglected in the consideration of mosquito ecology in the UK. Here, I sampled mosquitoes across 4 sites in Lincolnshire, east England, across 2 summers (April – July inclusive). 1026 mosquitoes were caught across 2 years, comprising 12 species, identified morphologically and confirmed genetically. Nine species (21 individuals; 5·8%; *n* = 363) had haemoparasite DNA in their salivary glands, with 2 lineages of *Plasmodium*, 3 of *Haemoproteus* and 1 of *Leucocytozoon* returning high-quality sequence. *Ochlerotatus detritus* is reported for the first time as a potential vector of *Plasmodium vaughani* (SYAT05), and *Ochlerotatus annulipes* and *Anopheles messeae* are reported for the first time as potential vectors of avian *Plasmodium* sp. (lineage LK06). Avian haemosporidians are diverse and widespread in UK birds and mosquitoes, and should be more widely considered in mosquito ecology in order to understand their complex transmission dynamics.

## Graphical abstract

**Figure:**
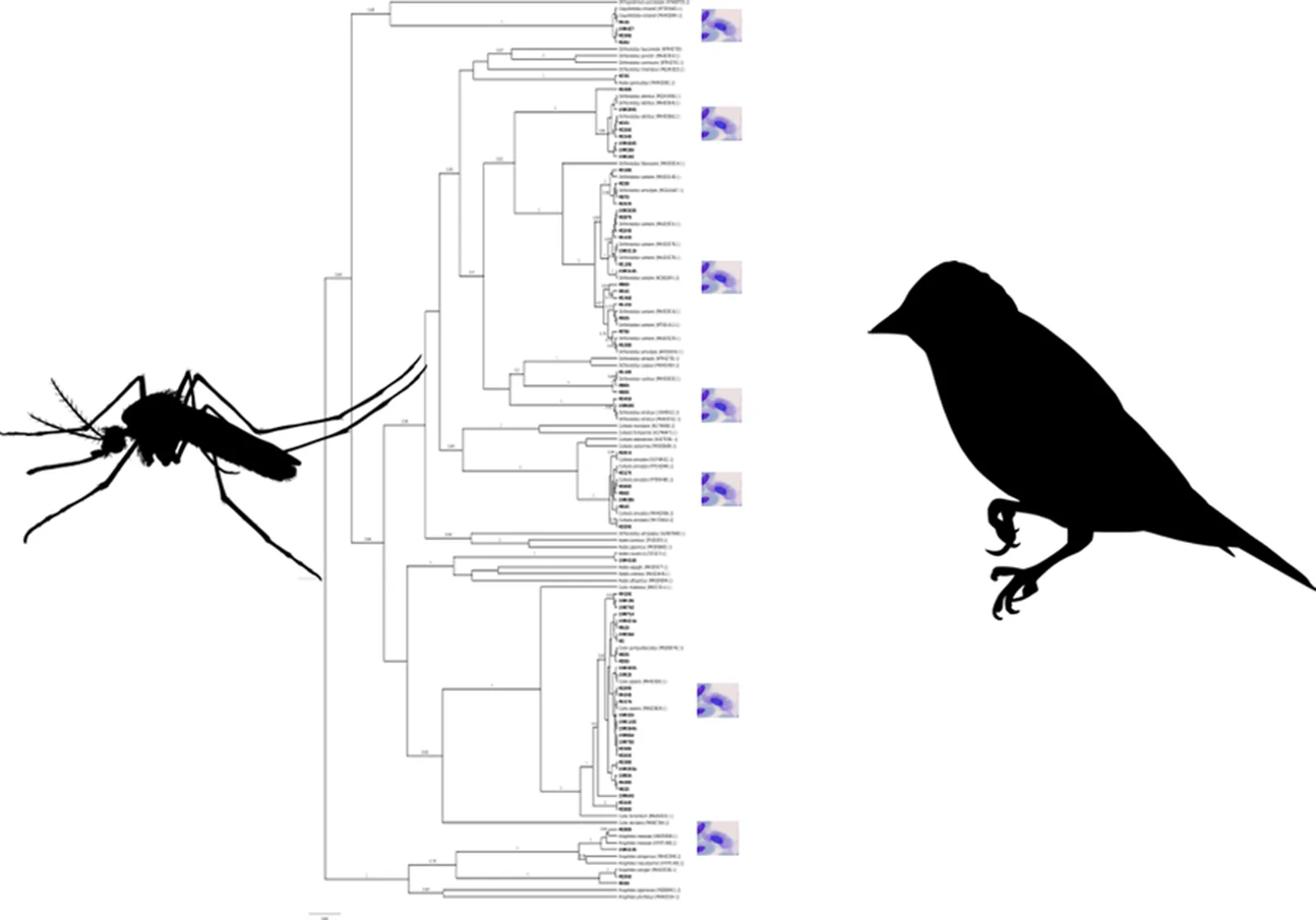

## Introduction

Avian haemoparasites (*Plasmodium* spp., *Haemoproteus* spp. and *Leucocytozoon* spp.) are cosmopolitan parasites found on every continent except Antarctica (Valkiūnas, 2005). They are well-documented to cause morbidity and mortality in naïve avian hosts (Grilo et al., 2016) but can also have impacts on physiology, reproduction and survival in hosts with which they have coevolved (Marzal et al., 2005, 20; Krams et al., 2013; Himmel et al., 2021). However, we still know remarkably little about their transmission dynamics. Despite wide research on the potential for UK mosquitoes to transmit a range of diseases pertinent to human health, their potential to transmit diseases of wildlife is largely overlooked (but see recent work on Usutu virus; Pilgrim et al., 2024).

Avian haemoparasites are incredibly diverse, with over 5300 distinct genetic lineages characterized at the cytochrome b barcoding region (Bensch et al., 2009; last accessed 30/03/2026). Haemoparasites sit on a continuum from species-specific to completely generalist in terms of their ability to infect different avian hosts (Doussang et al., 2021; Woodrow et al., 2023), but their associations with vectors are much less well-characterized (Gutiérrez-López et al., 2020a).

Haemoparasites are transmitted between birds through the bite of an infected dipteran. Generally, *Plasmodium* spp. are transmitted by mosquitoes (Culicidae), *Haemoproteus* by both biting midges (Ceratopogonidae; subgenus *Parahaemoproteus*) and flat flies (Hippoboscidae; subgenus *Haemoproteus*), and *Leucocytozoon* by blackflies (Simuliidae). The dipteran vector is also the definitive host for avian haemosporidians, in which sexual reproduction occurs, and studies suggest that the vector–parasite relationship may be just as specific as that of the intermediate avian host–parasite relationship (Gutiérrez-López et al., 2020a,b). For example, an experimental study identified that *Culex pipens* could transmit 2 lineages each of *Plasmodium relictum* and *P. cathemerium*, whereas *Aedes caspius* was not able to transmit lineages of either species (Gutiérrez-López et al., 2020a). However, *Cx. pipiens* infected by *P. relictum* showed reduced survival and transmission rate (Gutiérrez-López et al., 2020a).

Multiple factors are likely to influence the specificity of the vector–parasite relationship, not just the biological competence of the vector for parasite development. Even in competent vectors, the vector needs to encounter a competent host on multiple occasions: first for the vector to become infected and, following parasite development within the vector, subsequently in order for the vector to transmit parasite sporozoites to the next vertebrate host. Depending on the availability of competent hosts, and blood meal preferences of the vector, this may be more or less likely to occur in different ecological contexts.

Many studies examining parasite–vector associations use polymerase chain reaction (PCR) screening of the whole mosquito, or of the thorax within which the salivary glands reside (e.g. Kimura et al., 2010; Ferraguti et al., 2013). However, this can also amplify DNA from abortive infections, whereby the parasite develops oocysts within the mosquito mid-gut, but is not able to develop to the transmissible sporozoite stage (Žiegytė and Valkiūnas, 2014). The frequency of this occurrence in relation to the frequency of development of transmissible infections is unknown and thus, the confirmation of vector competence – or the presence of the parasite in the salivary glands, is essential to confirm transmission potential (Žiegytė and Valkiūnas, 2014).

Understanding parasite–vector relationships is crucial if we are to understand and evaluate infection and disease risk. Here, I take the first steps towards improving our understanding of avian malaria vectors in the UK by sampling mosquitoes at 4 farmland sites where the haemosporidian lineages of passerine bird communities have also been characterized (Woodrow et al., 2023; Armour et al., 2025, J.C. Dunn, unpublished data). First, mosquito community composition was assessed by identifying each mosquito to species using both morphological and molecular techniques, confirming the identity of each morphospecies through sequencing a section of the *cytochrome c oxidase* (*COI*) gene. Second, to identify vector–parasite relationships and identify competent vectors, salivary glands were dissected out from a subset of mosquitoes to test for the presence of parasite DNA (sporozoites) in salivary glands through PCR and sequencing of positive samples.

## Materials and methods

### Field sites and mosquito collection

Samples were collected from 4 field sites within 10 km of Lincoln, Lincolnshire, UK (Glentham: 53°31′ N, 0°22′ W; Potterhanworth: 53°11′ N, 0°25′ W; Eagle: 53°11′ N, 0°41′ W; North Carlton: 53°13′ N, 0°31′ W) between April and July (inclusive) during 2018 and 2019. All sites were surrounded by arable farmland, situated in small areas of scrub or woodland with or without water. The trapping sites at Glentham were situated around 3 medium-sized (4000–5400 m^2^) elevated freshwater reservoirs with reed beds and willow trees, surrounded by scrub (mostly common hawthorn *Crataegus monogyna* and blackthorn *Prunus spinosa*). Trapping sites at Potterhanworth were situated in a stand of mixed broadleaved woodland with some scrub understorey; the only water sources were small plastic trays of water supplied for game birds. Sites at Eagle were either in a stand of open woodland with a small freshwater pond (in 2018), or scrub (mostly hawthorn and bramble *Rubus fruticosus*) situated with 50 m of a stagnant shallow pool (mostly overgrown and shaded; in 2018 and 2019). The site at North Carlton consisted of a stand of mature woodland managed for game birds (with water sources in shallow plastic trays), with a stagnant pond (in permanent shade).

Traps were set at least once per month at each site. In 2018, only CO_2_- and light-baited CDC (Centers for Disease Control and Prevention) traps (BioQuip Products, Rancho Dominguez, CA, USA) were used to target host-seeking female mosquitoes, with 4 set per each capture session. In 2019, both CO_2_- and light-baited CDC traps and 2–4 gravid traps (John W Hock Co., Gainsville, FL, USA) per capture session baited with grain and hay-infused water were used to target host-seeking and ovipositing female mosquitoes, respectively. All traps were set before 19:00, left for 1 night and collected after 09:00 the next morning.

### Mosquito processing

Following capture, mosquito traps were stored at −20 °C for 10 min to humanely kill the mosquitoes. Mosquitoes were placed into individually numbered tubes and stored at 4 °C until they were processed (within 3 days of capture). Mosquitoes were identified morphologically using the MosKeyTool (Gunay et al., 2018). Salivary glands were dissected out where possible by removing the head of the mosquito with a sterile scalpel blade and gently pressing on the thorax to eject the salivary glands into a drop of sterile phosphate-buffered saline. Mosquitoes kept for longer than 24 h before attempting salivary gland dissections generally did not yield salivary glands because they had desiccated. Salivary glands were then severed using a sterile scalpel blade under ×100 magnification, transferred into 10 mL DNAzol™ (Chomczynski et al., 2018; Invitrogen, Carlsbad, California) and stored at −20 °C before DNA extraction. Abdomens, heads and thoraxes were stored separately, each in 10 mL DNAzol™ at −20 °C.

### DNA extraction and parasite screening

DNA was extracted from salivary glands using DNAzol™. Salivary glands were manually ground in 100 μL DNAzol™, and centrifuged for 10 min at 10 000 *g*. Then, 50 μL of 100% EtOH was added to the supernatant and incubated for 5 min to precipitate the DNA. The mixture was centrifuged at 10 000 *g* for 10 min, and the remaining pellet then washed twice with 1 mL 75% EtOH and centrifugation. The remaining ethanol was removed, and the DNA pellet was thoroughly dried before being resuspended in 100 μL DNAse-free water.

Each salivary gland (*n* = 363) was screened for avian haemoparasites using the forward primer UNIVF (5′-CAYATAYTAAGAGAAYTATGGAG-3′) in combination with each of UNIVR1 (5-GCATTATATCWGGATGWGNTAATGG-3′) and UNIVR2 (5′-ARAGGAGTARCATATCTATCWAC-3′) in 2 separate reactions (Drovetski et al., 2014), which detect all *Plasmodium* strains within our bird population as well as *Haemoproteus* and *Leucocytozoon* (J.C. Dunn, unpublished data) with an expected product length of 506–536 bp. Reactions were carried out in a 10 μL reaction volume containing 5 μL 2X Qiagen Multiplex PCR Buffer (Qiagen, Manchester, UK), 0·2 μL each forward and reverse primer (10 mM), 3·6 μL RNase-free water and 1 μL template DNA. A positive and negative control was included with each PCR run to ensure successful amplification and a lack of contamination, respectively. Parasite DNA within salivary glands occurs at low concentrations, so all negatives were rescreened once with each primer pair to minimize the occurrence of false negatives. The PCR protocol involved 15 min initial denaturation at 95 °C, followed by 42 cycles of denaturation at 94 °C for 30 s, annealing for 30 s at 54 °C for UNIVR1 and 52°C for UNIVR2, and 45 s extension at 72 °C. This was followed by a final terminal extension step at 72 °C for 10 min. All PCR protocols were carried out on a BioRad T100 Thermal Cycler (BioRad, Hercules, CA, USA). PCR products were subsequently visualized under UV light on a 1% agarose gel stained with GelRed (Cambridge Bioscience, Cambridge, UK).

In order to ensure sufficient DNA for sequencing, weak positive samples were reamplified as above, using 1 µL of PCR product in place of template DNA and the same primer set. Where primer dimer was present, samples were purified using a QIAquick PCR purification kit (Qiagen, Hilden, Germany) prior to being sent for sequencing in both directions by Macrogen Europe (The Netherlands). Samples without primer dimer were not purified before being sent for sequencing.

### Molecular confirmation of mosquito identification

Salivary gland DNA extractions from 1 to 3 samples of each morphotype were subject to PCR to amplify a 710 bp section of the mitochondrial *COI* gene using primers LCO1490 (5′-GGTCAACAAATCATAAAGATATTGG-3′) and HCO2198 (5′-TAAACTTCAGGGTGACCAAAAAATCA-3′) (Folmer et al., 1994). PCRs were carried out in 10 μL reaction volumes consisting of 5 μL 2X Qiagen Multiplex PCR Mastermix (Qiagen, Manchester, UK), 0·2 μL each of forward and reverse primers (10 mM), 1 μL template DNA and 3·6 μL RNase-free water. PCRs were carried out on a BioRad T100 Thermal Cycler (BioRad, Hercules, CA, USA), with a PCR protocol of an initial denaturation of 15 min at 95 °C, followed by 43 cycles of 94°C for 30 s, 52 °C for 60 s and 72 °C for 90 s, and a final extension step of 72 °C for 10 min. PCR products were sent for bidirectional sequencing by Macrogen Europe (The Netherlands).

Sequences were trimmed and the identity of mosquitoes was confirmed by comparison with sequences in GenBank using the NCBI-BLAST database (Altschul et al., 1997); the closest matching sequence was downloaded from GenBank to include in phylogenetic analysis. If not already present, a representative COI sequence from each species of mosquito known to be found in the UK (https://data.nbn.org.uk; last accessed 14/09/2021) was downloaded from GenBank to include in the alignment, where available.

A Generalized Time Reversible model with Gamma and Invariant sites was determined to be the best nucleotide substitution model for the trimmed alignment (565 bp) using jModeltest v 2.1.10 (Darriba et al., 2012) and Bayesian Information Criterion scores. Priors were defined using BEAUTi v1.10.4 (Drummond et al., 2016) including a strict clock and Yule speciation process (Gernhard, 2008). Bayesian phylogenetic trees were constructed using BEAST v1.10.4 (Suchard et al., 2018), with Markov Chain Monte Carlo simulations using 25 000 000 generations, sampled every 1000 generations with a 10% burn-in.

### Statistical analysis

Statistical analyses were carried out in R v. 3.6.1 (R Core Team, 2024). First, to test whether species abundance differed between month or trap types, chi-squared tests were conducted.

To test whether the prevalence of parasites detected in mosquito salivary glands was influenced by trap type, or differed spatiotemporally, a binomial generalized linear model with the presence or absence of parasites as the response variable was constructed. Fixed terms within the model were trap type (CDC or gravid; hand-caught mosquitoes were removed from analysis due to small sample size, *n* = 9), Year (2018 or 2019), Month (May, June or July) or site. The significance of each term was determined by comparison of the full model with and without the relevant term. Non-significant terms were removed in a stepwise fashion, until only significant terms (*P* < 0·05) remained.

## Results

### Mosquito community composition

**Figure 1.**
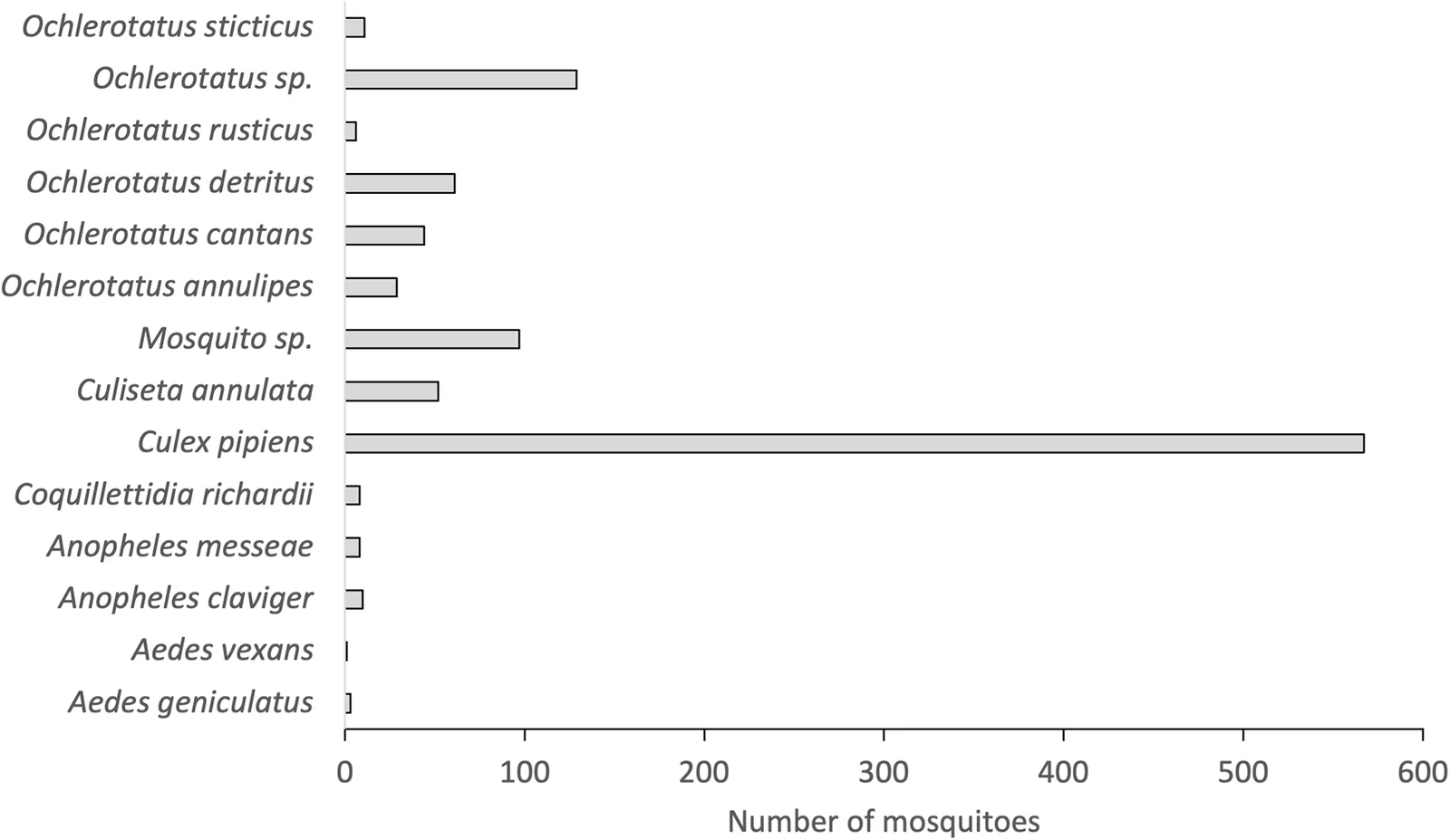
Community composition of mosquitoes caught from 4 sites in Lincolnshire, UK, in 2018 and 2019.

A total of 1026 mosquitoes were caught at 4 sites in 2018 and 2019. The presence of 12 species was identified morphologically and confirmed using molecular methods, with *Culex pipiens* the most common mosquito, accounting for 567 (55·26%) of all captures (Figure 1). *Culiseta annulata* was the next most common species (*n* = 52, 5·07%) followed by *Ochlerotatus detritus* (*n* = 61, 5·95%), *Oc. cantans* (*n* = 44, 4·29%), *Oc. annulipes* (*n* = 29, 2·83%) and *Oc. sticticus* (*n* = 11, 1·07%). *Anopheles claviger* (*n* = 10, 0·97%), *Coquillettidia richiardii* (*n* = 8, 0·78%), *Anopheles messeae* (*n* = 8, 0·78%), *Oc. rusticus* (*n* = 6, 0·58%), *Aedes geniculatus* (*n* = 3, *P* = 0·29%) and *Ae. vexans* (*n* = 1, *P* = 0·10%) comprised <1% of the mosquito community each. Moreover, 129 (12·57%) captures could only be identified to the *Ochlerotatus* genus level, and 97 (9·45%) captures could not be identified beyond family level.

Mosquito species differed in their capture rates between the 2 trap types during 2019, when both trap types were deployed (χ^2^_9_ = 220·41, *P* < 0·001). *Cx. pipiens* (92% of individuals in gravid traps; *n* = 379), *Oc. sticticus* (86%, *n* = 7) and *Cs. annulata* (60%; *n* = 10) were caught more often in gravid traps than in CDC traps. Gravid traps also caught *Cq. richiardii* (50%; *n* = 2), and *Oc. detritus* (4·9%; *n* = 36). Mosquito species also differed in their abundance patterns between months (χ^2^_18_ = 351·57, *P* < 0·001). *Cx. pipiens* and *Cs. annulata* were both found in low numbers in May, with abundance increasing through June and July. *Oc. rusticus* and *Oc. sticticus* were also found in low numbers in May, with *Oc. rusticus* reaching a peak in June and then found in low numbers again in July and *Oc. sticticus* not captured in June and then caught again in July. All other species, with the exception of *Oc. annulipes* and *Oc. cantans*, were caught in lower numbers in June than in July. Due to differing capture methods, abundance was not compared between years.

**Figure 2.**
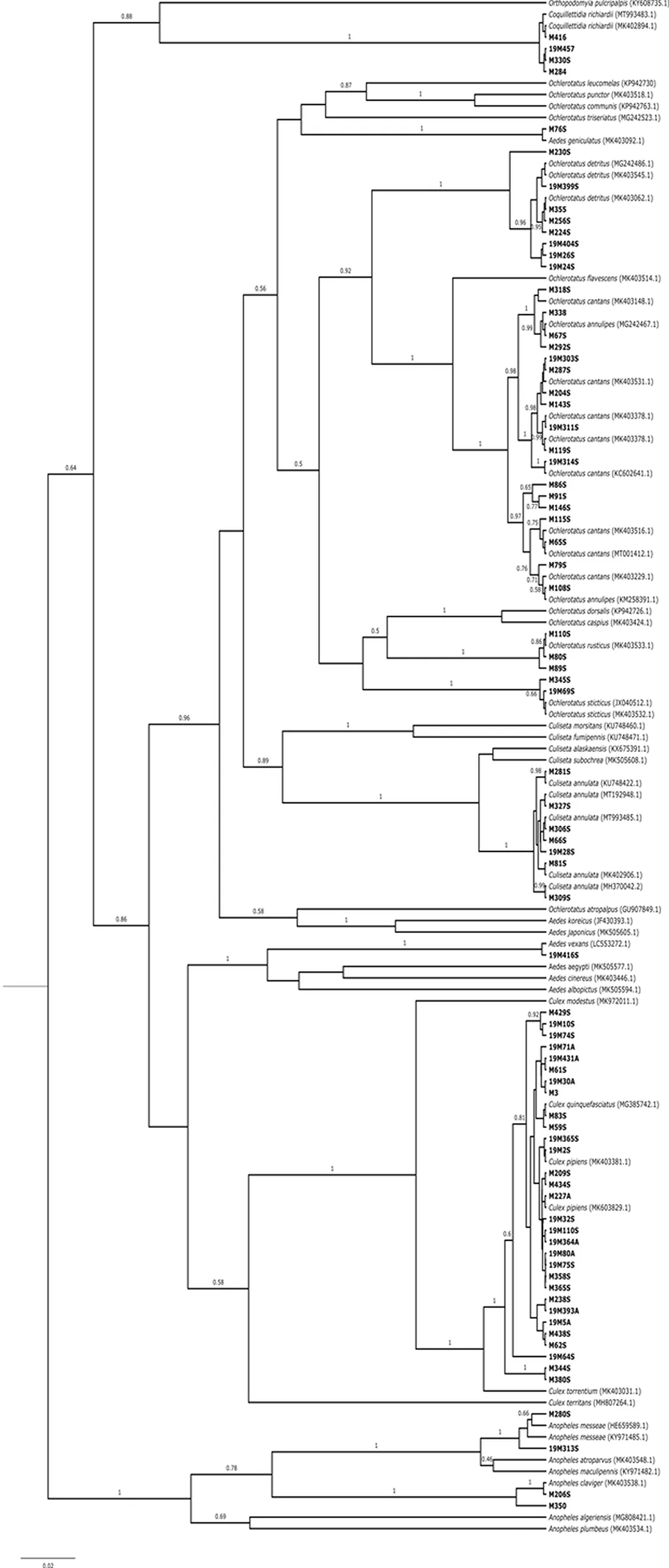
Bayesian phylogeny showing placement of mosquitoes sampled during this study in comparison to the closest matches on GenBank, and reference sequences from each mosquito species recorded within the UK from which sequence data were available. Samples from this study are highlighted in bold. The tree was created using an alignment of 565 bp of the *cytochrome oxidase 1* gene. Branch labels show posterior probabilities; only values ≥0·5 are displayed. Figure 2 long description.

Phylogenetic analysis of sequences generally generated good support for species-level clades (Figure 2), with the exception of *Oc. cantans* and *Oc. annulipes*, which formed a single combined clade.

### Haemoparasite prevalence in mosquito salivary glands

A total of 363 mosquito salivary glands were dissected and screened for haemoparasites across the 2 years of the study, of which 21 were positive for parasites (5·8% prevalence; Table 1). Parasite prevalence was not affected by trap type, and did not differ between month or sites (Table 2). Prevalence was marginally higher in 2018 (6·8%, *n* = 263) than in 2019 (2·2%, *n* = 91; Table 2), possibly due to different trapping techniques.

**Table 1.** Summary of the number of mosquitoes captured and salivary glands tested from each mosquito species, along with the number of samples testing positive for haemosporidian DNA Table 1 long description.

Species	Number captured	No. of salivary glands screened	No. of salivary glands +ve
*Aedes geniculatus*	3	3	0
*Aedes vexans*	1	0	0
*Anopheles claviger*	10	6	0
*Anopheles messeae*	8	5	1
*Coquillettidia richiardii*	8	5	1
*Culex pipiens*	567	190	6
*Culiseta annulata*	52	35	1
Mosquito sp.	97	8	0
*Ochlerotatus annulipes*	29	17	1
*Ochlerotatus cantans*	44	15	0
*Ochlerotatus detritus*	61	26	2
*Ochlerotatus rusticus*	6	4	0
*Ochlerotatus* sp.	129	39	0
*Ochlerotatus sticticus*	11	8	1

Details of positive samples and lineage IDs are provided in Table 3.

**Table 2.** Results from a binomial generalized linear model examining factors associated with parasite presence in mosquito salivary glands Table 2 long description.

Term	Estimate	SE	*df*	Dev	*p*
*Year*	*−1·185*	*0*·*756*	*1, 353*	*3·281*	*0·070*
Trap type	15·16	1211·23	1, 351	1·556	0·212
Month	–	–	2, 350	3·369	0·186
Site	–	–	3, 349	0·247	0·970

Statistics presented are from comparison of the final model with and without each term. Marginally significant (0·05 < *P* < 0·1) terms retained in the final model are highlighted in italics. Estimates ± 1 SE are included for 2-level factors.

Avian haemoparasite DNA was successfully sequenced from 16 individual mosquitoes from 9 species. Two lineages of *Plasmodium* (LK06 (*n* = 2) and SYAT05 (*n* = 1)) were detected in *Oc. annulipes, Oc. detritus* and *An. messeae* (Table 3). Five lineages of *Haemoproteus* (CARCHL01 (*n* = 3), DUNNO01 (*n* = 2), TUPHI01 (*n* = 2), EMCIR01 (*n* = 2) and SYAT02 (*n* = 1)) were detected in *Cx. pipiens, Oc. detritus, Cq. richiardii, Cs. annulata, An. claviger* and *An. messeae*, although all sequences matching DUNNO01 and SYAT02 were poor quality. Three lineages of *Leucocytozoon* were detected in *Cx. pipiens, Oc. detritus* and *Oc. sticticus*, although only 1 of these (PARUS11 (*n* = 1)) could be identified with confidence due to short sequence length (PARUS84/PARUS18 (*n* = 1)) or poor quality sequence (AEMO01 (*n* = 1)).

**Table 3.** Salivary glands testing positive for the presence of haemoparasite DNA Table 3 long description.

Sample ID	Trap type	Date	Site	Species	Parasite lineage	Parasite genus	Sequence length (bp)	% Similarity
M3	CDC	06/06/2018	North Carlton	*Culex pipiens*	–			
M83	CDC	21/06/2018	Eagle	*Culex pipiens*	DUNNO01*	*Haemoproteus*	405	94
M146	CDC	26/06/2018	North Carlton	*Ochlerotatus annulipes*	–			
M215	CDC	09/07/2018	Moorlands	*Culex pipiens* (morphological)	PARUS84 or PARUS18	*Leucocytozoon*	419	99
M225	CDC	09/07/2018	Moorlands	*Culex pipiens* (morphological)	CARCHL01*	*Haemoproteus*	473	97
M230	CDC	09/07/2018	Moorlands	*Ochlerotatus detritus*	CARCHL01	*Haemoproteus*	446	100
M235	CDC	09/07/2018	Moorlands	*Culex pipiens*	TUPHI01	*Haemoproteus*	439	100
M256	CDC	09/07/2018	Moorlands	*Ochlerotatus detritus*	PARUS11	*Leucocytozoon*	419	100
M259	CDC	09/07/2018	Moorlands	*Culex pipiens* (morphological)	EMCIR01	*Haemoproteus*	479	100
M284	CDC	17/07/2018	North Carlton	*Coquillettidia richiardii*	TUPHI01	*Haemoproteus*	422	100
M287	CDC	17/07/2018	North Carlton	*Ochlerotatus cantans*	–			
M292	CDC	17/07/2018	North Carlton	*Ochlerotatus annulipes*	LK06	*Plasmodium*	383	100
M306	CDC	17/07/2018	North Carlton	*Culiseta annulata*	DUNNO01*	*Haemoproteus*	465	95
M338	Hand	18/07/2018	North Carlton	*Ochlerotatus annulipes*	–			
M345	CDC	20/07/2018	Eagle	*Ochlerotatus sticticus*	AEMO01*	*Leucocytozoon*	308	99
M350	CDC	20/07/2018	Eagle	*Anopheles claviger*	SYAT02*		436	97
M355	CDC	24/07/2018	Moorlands	*Ochlerotatus detritus*	SYAT05	*Plasmodium*	475	100
M357	CDC	24/07/2018	Moorlands	*Anopheles messeae* (morphological)	LK06	*Plasmodium*	478	100
M363	CDC	25/07/2018	Glentham	*Anopheles messeae*	CARCHL01	*Haemoproteus*	445	100
19M11	Gravid	20/05/2019	North Carlton	*Culiseta annulata* (morphological)	–			
19M74	Gravid	10/07/2019	Eagle	*Culex pipiens*	EMCIR01	*Haemoproteus*	466	100

Mosquito ID is through analysis of PCR sequence data and comparison to GenBank unless stated as morphological. ‘–’ in the parasite lineage column indicates that sequencing of the PCR product was not successful for this sample; * indicates a poor quality sequence. Where 2 parasite lineages are specified, the sequence obtained matches both at the same % similarity over the length of the sequence.

## Discussion

Twelve species of mosquito were detected during this study, confirmed using molecular methods and supported by phylogenetic analysis. This diversity is consistent with other mosquito surveillance studies within the UK (Medlock and Vaux, 2015). Haemoparasite DNA was confirmed in 5·8% of mosquito salivary glands, with good-quality sequence identifying 2 *Plasmodium* lineages (SYAT05 and LK06), 3 *Haemoproteus* lineages (EMCIR01, TUPHI01 and CARCHL01) and 1 *Leucocytozoon* lineage (PARUS 11); all of these lineages are known from the local bird community (J.C. Dunn et al., unpublished data).

To date, 36 mosquito species have been recorded in the UK (Chandler, 2023). The diversity recorded here is comparable with other mosquito surveys in the east of England (e.g. Medlock and Vaux, 2015). For example, Medlock and Vaux (2015) recorded 15 mosquito species in the Cambridgeshire Fens, approximately 120 km south of Lincoln, and 19 mosquito species were recorded in a broader study of wetland habitats across the UK (Medlock et al., 2024). The species composition from Lincolnshire was largely similar to that of Medlock and Vaux (2015), with the notable exception of *Cx. pipiens*, which was the most common species recorded in Lincolnshire but present only in very low numbers in Cambridgeshire (Medlock and Vaux, 2015), and in a broader survey of UK wetlands (Medlock et al., 2024). *Cx. pipiens* is the species of mosquito best-known for transmitting avian malaria across Europe (e.g. Inci et al., 2012; Garrigós et al., 2024), as well as West Nile Virus and lymphatic filariasis, among others, and is 1 of the most intensively studied vector species across temperate regions (Haba and McBride, 2022). This finding is likely explained by sampling methods: the gravid traps used in Lincolnshire attracted very high numbers of *Cx. pipiens*, whereas Medlock and Vaux (2015) acknowledge that the Mosquito Magnet traps used in Cambridgeshire – and across the UK wetlands – are likely to under-sample ornithophilic species such as *Cx. pipiens* (Medlock and Vaux, 2015). Interestingly, *Aedes cinereus* was not identified in Lincolnshire, whereas it was highly abundant in some habitats in Cambridgeshire (Medlock and Vaux, 2015) and present at multiple wetland sites across the UK (Medlock et al., 2024).

*Culex torrentium* was not identified in Lincolnshire, which is in line with previous surveys of *Culex* sp. distribution (Widlake et al., 2025). However, it should be noted that *Cx. pipiens* and *Cx. torrentium* are difficult to differentiate morphologically (Medlock et al., 2024), so it is possible that *Cx. torrentium* may have been present in small numbers and overlooked during morphological identification. One *Ae. vexans* individual was identified in Lincolnshire, with identification confirmed genetically. *Ae. vexans* is generally found in low numbers in the UK, although recent exceptions have identified isolated populations at relatively high densities (Vaux et al., 2021; Johnston et al., 2023).

Phylogenetic analysis provided good support for species identification, with all species forming monophyletic clades, with 2 exceptions. First, a reference sequence ostensibly from *Cx. quinquefasciatus* was included because it was the closest BLAST match on GenBank for several species morphologically identified as *Cx. pipiens*. This seems to suggest a potential identification error for the reference sequence (Cane et al., 2020), given the absence of *Cx. quinquefasciatus* from the British list of dipteran species (Chandler, 2023) and the conclusion of Cane et al. (2020) that this *Cx. quinquefasciatus* was closely related to *Cx. pipiens* (Cane et al., 2020). Second, *Oc. annulipes* and *Oc. cantans* are found within a single clade, highlighting the difficulties of separating these 2 species morphologically (Medlock et al., 2024).

The seasonal dynamics in mosquito abundance found in Lincolnshire mirror those found in other studies. The 4-month sampling period does not encompass the full period during which mosquitoes are active within the UK, so the second peak in abundance in the autumn reported by many studies (Ferraguti et al., 2013; Medlock and Vaux, 2015) was not observed here. However, our observation of a general increase in abundance from April to July largely corresponds with findings elsewhere (e.g. Ferraguti et al., 2013).

The 5·8% prevalence of avian haemoparasite DNA in mosquito salivary glands is in line with most other studies. For example, Köchling et al. (2023) found a 5·2% minimum infection rate (MIR) in mosquitoes in Germany and Schoener et al. (2017) found a 5% MIR in *Cx. pipiens, Cx. torrentium* and *Cx. pipiens* x *Cx. molestus* hybrids in Austria (Schoener et al., 2017) when working with pooled mosquito samples. In contrast, Ferraguti et al. (2013) found a lower prevalence of 0–3·2% in mosquito species in southern Spain whereas Lalubin et al. (2013) found 15·4% of *Cx. pipiens* mosquitoes in Switzerland to be positive for *Plasmodium*.

Only 2 lineages of *Plasmodium* were identified from mosquitoes in Lincolnshire, both of which are known from multiple avian hosts within the local bird communities (J.C. Dunn et al., unpublished data). SYAT05 is a widespread *Plasmodium vaughani* lineage that has previously been identified from *Cx. pipiens* in multiple European countries (Glaizot et al., 2012; Lalubin et al., 2013; Synek et al., 2013; Zélé et al., 2014; Martínez-de la Puente et al., 2015; Schoener et al., 2017; Kapustová et al., 2025), Turkey (Inci et al., 2012), Japan (Kim and Tsuda, 2010) and the US (Kimura et al., 2010), *Ae. albopictus* in Italy (Martínez-de la Puente et al., 2015), *Cx. restuans* in the US (Kimura et al., 2010), *Cx. modestus* and *Cx. perexiguus* in Spain (Ferraguti et al., 2013), *Cx. theileri* in Portugal and Spain (Ventim et al., 2012; Ferraguti et al., 2013), *Cx. torrentium* in Germany (Köchling et al., 2023) and from 2 species of biting midge in Lithuania (Chagas et al., 2022, 2024). SYAT05 has not previously been recorded in *Oc. detritus* so it may be that this is a new vector species for this parasite lineage. LK06 (*Plasmodium* sp.) has not previously been isolated from any vector species, so *Oc. annulipes* and *An. messeae* are the first reported potential vector species for this parasite lineage; *An. messeae* is known as a vector of human malaria (Brusentsov et al., 2023) but to my knowledge neither of these mosquito species have previously been associated with avian malaria transmission (MalAvi Vector data table, https://wimanet-science.github.io/web/malavi/tables/, accessed 10/04/2026; Bensch et al., 2009). Further experimental work is needed to confirm these species as vectors of these lineages. Interestingly, SGS1 (*Plasmodium relictum*), LINN1 (*Plasmodium matutinum*) and TURDUS1 (*Plasmodium circumflexum*) are also common within the local bird community (J.C. Dunn et al., unpublished data) but were not identified from mosquitoes in this study. Schoener et al. (2017) found SYAT05 to be common in *Cx. pipiens* early in the year in Austria, but this lineage was replaced by SGS1 and LINN1 later in the year, so it is possible that the sampling season in Lincolnshire did not overlap with peak transmission of these lineages.

The finding of DNA from multiple *Haemoproteus* lineages in mosquito salivary glands is of note, because mosquitoes are not thought able to transmit *Haemoproteus* parasites (Valkiūnas, 2005). The isolation of salivary glands from the thorax prior to PCR should limit contamination from other mosquito tissues, although this cannot be ruled out entirely. Multiple studies have previously reported associations between mosquitoes and *Haemoproteus* (Ishtiaq et al., 2008; Njabo et al., 2011; Ferraguti et al., 2013; Mora-Rubio et al., 2023; Kapustová et al., 2025), but these studies generally conduct PCR on the whole mosquito rather than just the salivary glands. Experimental infections of mosquitoes with *Haemoproteus* show contrasting results, although none has confirmed mosquito transmission of this parasite genus. Gutiérrez-López et al. (2016) found an absence of *Haemoproteus* DNA in the saliva, or the head–thorax of experimentally infected *Cx. pipiens*, whereas Valkiūnas et al. (2013) found *Haemoproteus* ookinetes in head, thorax and abdomen of experimentally infected mosquitoes, but with no sporozoites in salivary glands. Crucially, this meant that *Haemoproteus* DNA was detectable throughout mosquito (Valkiūnas et al., 2013). Subsequent experiments identified that *Haemoproteus* is capable of killing *Ae. caspius* individuals without the development of ookinetes (Valkiūnas et al., 2014). These experiments highlight both the potential impact of *Haemoproteus* infections on bird-biting mosquitoes in the wild, but also the variation in these impacts. Given the likely species-specificity of the vector–parasite relationship, and the long-lasting nature of *Haemoproteus* DNA in wild mosquitoes as demonstrated by its finding in both fed and unfed mosquitoes both in this study and in southern Spain (Ferraguti et al., 2013), further experiments are needed to test whether mosquitoes are capable of transmitting *Haemoproteus* using combinations of vector species and parasite lineages observed in wild-caught mosquitoes.

However, given current knowledge, it seems more likely that the findings of both *Haemoproteus* and *Leucocytozoon* DNA in mosquito salivary glands most likely represent remnant and degraded DNA from an ingested blood meal, rather than confirming the presence of viable sporozoites in the salivary glands and vector competence for mosquito transmission of these parasite lineages. This is further supported by the short length of many of the *Haemoproteus* lineages isolated. The *Haemoproteus* lineages CARCHL01, DUNNO01, TUPHI01, EMCIR01 and SYAT02 are all present in the local bird community (Woodrow et al., 2023). Ideally microscopic examination of 1 salivary gland should be conducted to confirm the presence of viable sporozoites in conjunction with PCR of the other to identify parasite lineage (Valkiūnas, 2011).

Avian *Plasmodium* and *Haemoproteus* are common parasites of birds that are prevalent globally, including in the UK. Both these parasite genera have been shown to detrimentally impact mosquito survival (Žiegytė and Valkiūnas, 2014; Martínez-de la Puente et al., 2018; Adams et al., 2021) but have been neglected in consideration of mosquito ecology in the UK (Medlock and Snow, 2008). Previous examination of vector transmission of avian disease in the UK has focussed on Usutu virus (Pilgrim et al., 2024) and the potential for transmission of arboviruses (Blagrove et al., 2016; Brugman et al., 2018); here I highlight that avian malaria and other blood parasites are also widespread, native and diverse vector-transmitted parasites within the UK avifauna (Cosgrove et al., 2008; Dunn et al., 2013, 2017; Woodrow et al., 2023; Lebeau and Dunn, 2024), and ones that are deserving of further research to understands their complex transmission dynamics.

## Figure 2 Long description

The diagram is a vertical Bayesian phylogenetic tree showing the placement of mosquito samples from a study compared to GenBank matches and UK reference sequences. The tree is constructed using 565 base pairs of the cytochrome oxidase 1 gene. Branches are labeled with posterior probabilities, only displaying values greater than or equal to 0.5. The tree begins at the top with a single branch that splits into multiple branches, each representing different mosquito species. Samples from the study are highlighted in bold. The tree shows species-level clades with good support, except for Oc. cantans and Oc. annulipes, which form a combined clade. The branches extend downward, with various splits and merges, indicating evolutionary relationships among the species. Each branch ends with a label indicating the species name and sequence identifier.

Navigate back to Figure 2.

## Table 1 Long description

The table lists mosquito species with counts captured, salivary glands screened, and how many screened glands tested positive for haemosporidian DNA. Culex pipiens was by far the most abundant with 567 captured, 190 glands screened, and 6 positives, the highest positive count. Across all species, 361 salivary glands were screened and 13 were positive. Additional positives occurred in Ochlerotatus detritus with 2, and single positives in Anopheles messeae, Coquillettidia richiardii, Culiseta annulata, Ochlerotatus annulipes, and Ochlerotatus sticticus. Several species had no positives despite screening, including Aedes geniculatus, Anopheles claviger, Ochlerotatus cantans, Ochlerotatus rusticus, Mosquito sp., and Ochlerotatus sp. Aedes vexans had 1 captured but no salivary glands screened, so no inference about positivity can be made for that species. Because screening effort varies widely by species, comparisons of positivity should consider the different numbers of glands tested.

Navigate back to Table 1.

## Table 2 Long description

The table reports results from a binomial regression assessing which factors are associated with parasite presence in mosquito salivary glands. Year has a negative estimate of minus 1.185 with standard error 0.756, degrees of freedom 1 and 353, deviance 3.281, and p value 0.070, indicating a borderline association. Trap type has an estimate 15.16 with a very large standard error 1211.23, degrees of freedom 1 and 351, deviance 1.556, and p value 0.212, suggesting no clear evidence of an effect and high uncertainty. Month is tested as a multi-level term with degrees of freedom 2 and 350, deviance 3.369, and p value 0.186, not statistically significant. Site is also a multi-level term with degrees of freedom 3 and 349, deviance 0.247, and p value 0.970, showing little evidence of differences by site. Overall, none of the terms meet a conventional 0.05 threshold, and the year effect is the closest to significance.

Navigate back to Table 2.

## Table 3 Long description

The table lists mosquito salivary-gland samples that tested positive for haemoparasite DNA, with trap type, collection date and site, mosquito species, parasite lineage and genus, and sequence match details. There are 21 samples from May 2019 and June to July 2018, mostly collected with CDC traps, plus one hand-caught and two gravid-trap samples. Haemoproteus is the most common genus, appearing in eight samples across several mosquito species including Culex pipiens, Ochlerotatus detritus, Coquillettidia richiardii, Culiseta annulata, and Anopheles messeae. Plasmodium occurs in three samples, all with complete similarity matches, and Leucocytozoon occurs in three samples, including one lineage that could not be distinguished between two named lineages at the same similarity. Most successful sequences show complete similarity matches, while a few are lower and marked as poor quality, including DUNNO01 and CARCHL01 and AEMO01 and SYAT02. Several samples have a lineage recorded as not obtained, indicating sequencing did not succeed for those positives, so genus and match metrics are missing for those rows. Sequence lengths for successful results range from just over 300 base pairs to just under 500 base pairs, with most in the low to mid 400s.

Navigate back to Table 3.
